# Broadening the *PHIP*-Associated Neurodevelopmental Phenotype

**DOI:** 10.3390/children11111395

**Published:** 2024-11-17

**Authors:** Giulia Pascolini, Giovanni Luca Scaglione, Balasubramanian Chandramouli, Daniele Castiglia, Giovanni Di Zenzo, Biagio Didona

**Affiliations:** 1 Genetic Counselling Unit, Istituto Dermopatico dell’Immacolata, IDI-IRCCS, Via dei Monti di Creta 104, 00167 Rome, Italy; 2 Bioinformatic Unit, Istituto Dermopatico dell’Immacolata, IDI-IRCCS, 00167 Rome, Italy; g.scaglione@idi.it; 3 Super Computing Applications and Innovation, Department High Performance Computing (HPC), CINECA, 40033 Bologna, Italy; b.chandramouli@cineca.it; 4 Molecular and Cell Biology Laboratory, Istituto Dermopatico dell’Immacolata, IDI-IRCCS, 00167 Rome, Italy; d.castiglia@idi.it (D.C.); g.dizenzo@idi.it (G.D.Z.); 5 Rare Diseases Center, Istituto Dermopatico dell’Immacolata, IDI-IRCCS, 00167 Rome, Italy; b.didona@idi.it

**Keywords:** *PHIP*, neurodevelopment, Chung–Jansen syndrome (CHUJANS), Pitt–Hopkins syndrome (PTHS)-like phenotype, abnormal skin appendages, teeth anomalies

## Abstract

Background: Monoallelic damaging variants in *PHIP* (MIM*612870), encoding the Pleckstrin Homology Domain Interacting Protein, have been associated with a novel neurodevelopmental disorder, also termed Chung–Jansen syndrome (CHUJANS, MIM#617991). Most of the described individuals show developmental delay (DD)/intellectual disability (ID), obesity/overweight, and variable congenital anomalies, so the condition can be considered as an ID–overweight syndrome. Case Description: We evaluated a child presenting with DD/ID and a craniofacial phenotype reminiscent of a Pitt–Hopkins syndrome (PTHS)-like condition. We performed a clinical exome analysis on his biological sample, as well as an in silico prediction of the obtained data. At the same time, we interrogated the DeepGestalt technology powered by Face2Gene (F2G), using a frontal image of the proband, and clinically reviewed the earlier CHUJANS patients. In this child, we found a novel *PHIP* pathogenetic variant, which we corroborated through a protein modeling approach. The F2G platform supported the initial clinical hypothesis of a PTHS-like condition, while the clinical review highlighted the lack of the main frequent CHUJANS clinical features in this child. Conclusions: The unusual clinical presentation of this novel patient resembles a PTHS-like condition. However, a novel variant in *PHIP* has been unexpectedly detected, expanding the phenotypic spectrum of CHUJANS. Notably, PTHS (MIM#610954), which is a different ID syndrome caused by heterozygous variants in *TCF4* (MIM*610954), is not classically considered in the differential diagnosis of CHUJANS nor has been cited in the previous studies. This could support other complex diagnoses and invite further patients’ descriptions.

## 1. Introduction

The *PHIP* gene (MIM*612870), belonging to the bromodomain family III, encodes the full length Pleckstrin Homology Domain Interacting Protein/DDB1- and CUL4-associated factor 14 (PHIP/DCAF14), which is linked to neurodevelopment [1]. The full-length PHIP/DCAF14 protein product contains eight C-terminal WD40 repeats, an insulin receptor substrate-1 (IRS-1) domain, two bromodomains, and a nuclear localization signal [1].

So far, heterozygous *PHIP* perturbations have been found in intellectually disabled individuals, mainly presenting with overweight/obesity, behavioral features, craniofacial (high forehead, full eyebrows/synophrys, fleshy ears, upturned nose with thick alae nasi, long philtrum, and thin lips) and limb defects (tapered fingers, clinodactyly, and syndactyly of the second and third toes). The clinical core is consistent with an intellectual disability (ID)–overweight disorder, known as Chung–Jansen syndrome (CHUJANS; MIM#617991) [1]. Other features, including vision and balance problems, hypotonia, fatigue, urogenital defects, constipation, and hypo/hyperpigmentation can be recognized [1].

In the previous reports, stop-gained, frameshift, missense, and splice variants have been identified. These fell within the WD40 repeat domain, the bromodomain, and the IRS-1 PH binding domain [1].

Less than 10 papers including craniofacial descriptions are available to date, pointing out the limited amount of clinical information and the need of additional phenotypic descriptions to better define the condition.

Here, we describe a novel patient with an unusual phenotype that does not resemble the typical *PHIP*-associated syndrome (CHUJANS). Indeed, his craniofacial anomalies initially addressed the molecular study for a Pitt–Hopkins syndrome (PTHS)-like condition with negative results. It is known that PTHS (MIM#610954), which represents a well-defined ID disorder, is associated with perturbations in the *TCF4* gene (MIM*610954) [2,3]. The main PTHS clinical characteristics encompass neurodevelopmental delay, distinctive craniofacial anomalies (narrow forehead, thin lateral eyebrows, wide nasal bridge with flared alae, full cheeks, wide mouth with full lips, cupid bow upper lip, and thickened/overfolded helices) and breathing disorders (intermittent hyperventilation and apnea) [2,3,4]. Myopia, constipation, hand anomalies, and unstable gait can also be diagnosed [4].

In this patient, a previously unpublished deleterious variant in *PHIP* was exceptionally disclosed using a clinical exome approach.

**Figure 1:**
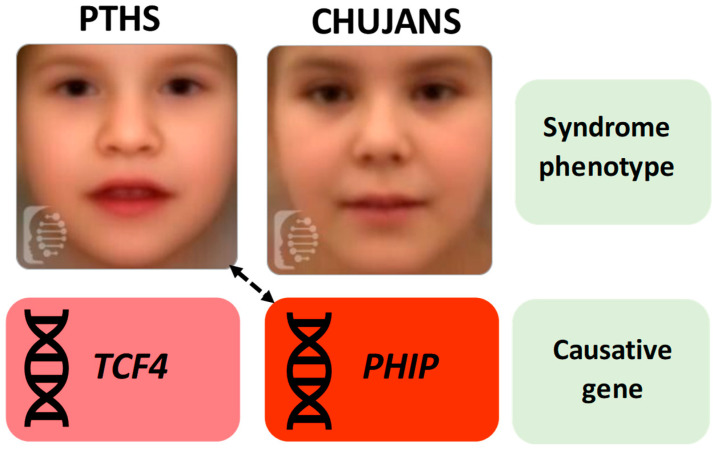
Face masks of PTHS and CHUJANS (acquired from F2G) and respective causative genes. In this patient, a connection (indicated by the arrow) between the PTHS clinical spectrum, mainly caused by *TCF4* perturbations, and *PHIP*, associated with CHUJANS, is recognizable.

Here, the clinical and molecular spectrum of CHUJANS is further expanded, describing a patient with an uncommon clinical presentation, resembling a PTHS-like disorder and suggesting a possible connection between the PTHS-like phenotypes and *PHIP* anomalies (Figure 1).

## 2. Case Description

### 2.1. Clinical Findings

This study was conducted according to the Helsinki declaration. Informed consent for the use of the clinical data and images was obtained from the patient’s guardians.

Information about the origin family, as well as the prenatal, neonatal, and infantile periods, was unavailable because the patient was adopted. 

**Figure 2:**
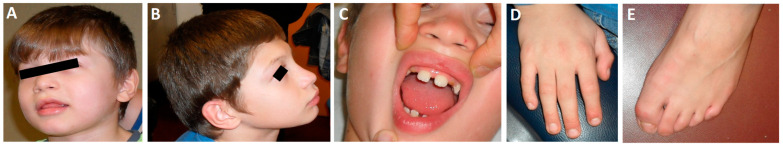
(**A**,**B**) Frontal and lateral views of the craniofacial phenotype of this patient at the ages of 4 years and 5 months and 9 years and 9 months, respectively. Note: wide mouth with full lips, wide nasal tip with flared alae nasi, and full and prominent cheeks. (**C**) Widely spaced teeth. (**D**,**E**) Extremities phenotype, consisting of clinodactyly of 5th finger and hands brachydactyly (**D**), broad halluces with camptodactyly, partial 2nd–3rd toe overriding, bilateral 4th–5th clinodactyly in his feet, and small nails on the 5th toes (**E**).

A patient aged 9 years and 9 months was genetically evaluated for global developmental delay, craniofacial dysmorphisms, and some cutaneous and appendage features, including very clear skin and abnormal teeth (Figure 2A–C).

He had been diagnosed with moderate ID (ICD10 F71) and severe attention deficit (ICD10 F90.0) since early infancy. Infantile neuropsychiatric evaluation (at the age of 7 years and 6 months) documented motor clumsiness with a tendency to walk on pointes; poorly purposed and confused exploration of the surrounding environment; poor interaction with adults and peers; the absence of communicative intent, except for limited primary needs; and the inability to perform daily acts. In addition, he requires special school support. 

At his last genetic evaluation, his weight was 30 kg (44.17 percentile), his height was 141 cm (74.42 percentile), and his occipitofrontal circumference (OFC) was 49 cm (1 percentile). 

The craniofacial phenotype resembled a PTHS-like condition, whereby he showed convergent strabismus, a distinctive nose conformation with a wide nasal tip and flared nasal alae, a wide mouth with full lips, a cupid bow upper lip, and full and prominent cheeks (Figure 2A,B and Table 1). Widely spaced teeth were also observed (Figure 2C). Bilateral clinodactyly of the 5th finger, brachydactyly, broad halluces with camptodactyly, partial 2nd–3rd toe overriding, and bilateral 4th–5th clinodactyly in his feet were identifiable, as well as small nails on the 5th toes (Figure 2D and Table 1). 

His language was poorly developed; he only answered questions with “yes” and “not” and was not cooperative during the clinical examination. 

### 2.2. Molecular Data

Previous standard karyotyping, cytogenomic analysis at 200 Kb resolution, targeted sequencing for PTHS and overlapping disorders, MLPA analysis of TCF4, and MS-MLPA analysis of the 15q11q13 locus were negative.

A comprehensive genomic profile of the proband using a next-generation sequencing (NGS) approach on Illumina NextSeq500 (Illumina, San Diego, CA, USA) was conducted. By using the TruSight One Expanded (Illumina) panel, we targeted the exonic regions of 6704 target genes with known disease-associated mutations. Variant filtering, annotation, and prioritization have been gained with a local java version of Exomiser (https://www.nature.com/articles/nprot.2015.124, accessed on 20 March 2024). Gene–disease and variant–disease associations were obtained using the HPO relevant features of the proband.

The clinical exome analysis disclosed the novel heterozygous variant c.1285A>G (p.Met429Val) in the *PHIP* gene (NM_017934.7).

The parental origin was not verifiable since he was adopted. Earlier reports described parentally inherited variants, as well as nucleotide changes, without an ascertained familiar transmission.

The variant, which was not previously published (although there is a single submission in ClinVar without an associated phenotype; Variation ID:1319725), can be classified as likely pathogenic (class IV), according to the ACMG guidelines (PM2, PP3, and PP5). 

### 2.3. Protein Modeling

**Figure 3:**
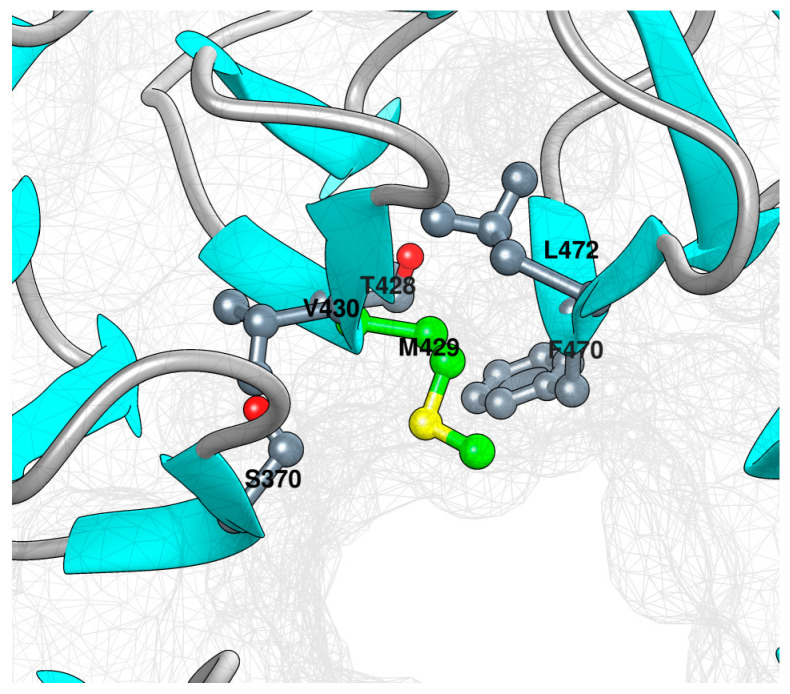
Structural model of WD40. The protein is depicted as a ribbon along the vdW surface (in gray).

In silico prediction was carried out through the alphafold model for WP40 [5] b with a prediction confidence score >90. With this approach, we assessed the impact of the mutation of site M429 and specifically the local interference it may produce (Figure 3). The alphafold model for WP40 showed that M429 in the WD6 repeat was sufficiently buried and extends towards the central cavity (Figure 3). The examination of nearby residues highlighted the methionine–aromatic interaction (M429-F470, Figure 3). Such non-covalent interactions, which are both abundant in proteins and known to be important for structural stability and function, may be impaired in the M429V phenotype [6,7].

### 2.4. DeepGestalt Analysis

To further outline the distinctive facial appearance of the present patient, we used the DeepGestalt technology powered by Face2Gene, (F2G, www.face2gene.com, accessed on 24 April 2024; Boston, MA, USA), uploading frontal portraits without clinical information.

The DeepGestalt technology (version DG 26.1.0) is an artificial intelligence (AI) tool for the facial analysis of genetic disorders. Using an input photo in the system, which is first pre-processed, it is possible to achieve facial and landmark detection, as well as alignment. The input image is cropped into facial regions, which are fed into a Deep Convolutional Neural Network (DCNN) to obtain a softmax vector, indicating its correspondence to each syndrome in the model. The output vectors of all regional DCNNs are then aggregated and sorted to gain a final ranked list of genetic syndromes (the 30 syndrome matches displayed in F2G) [8]. 

**Figure 4:**
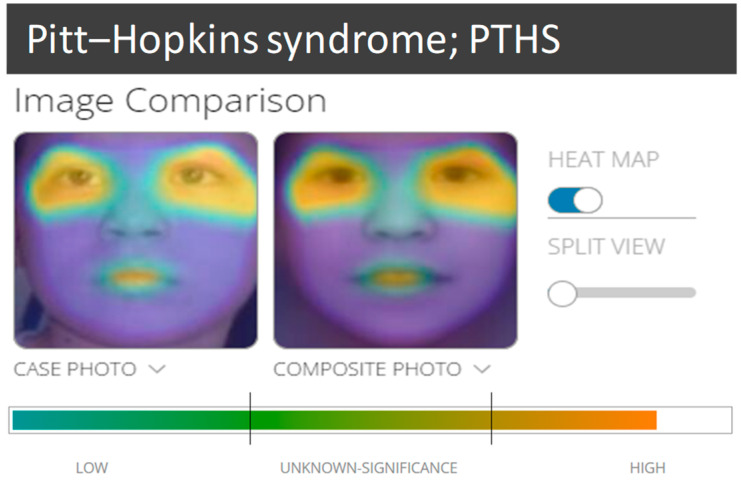
Overlapping facial regions with PTHS elaborated by F2G for the patient. The D-score for evaluating the degree of craniofacial dysmorphisms in this child is shown.

In this patient, the DeepGestalt analysis identified PTHS among the first eight most ranked syndromes, only analyzing his frontal portrait, without clinical information (Figure 4). Of note, CHUJANS did not appeared in the list of the 30 possible diagnoses, although the system is trained for its recognition. The most overlapping regions of similarity between our patient and PTHS were the eyes and the oral region (Figure 4). 

The craniofacial impairment of this patient was also confirmed by the D-Score of F2G, a new index of the degree of dysmorphic signs (Figure 4) [9]. 

### 2.5. Clinical Review and Comparison with the PHIP-Related Phenotype and PTHS Clinical Score

Moreover, a detailed clinical review of previous CHUJANS patients with reported craniofacial features was achieved (Table 2). 

We also compared the main features of our proband with those established for PTHS clinical diagnosis (Table 1) [4].

From a comparison with already described patients with CHUJANS, for whom there were available details about the face morphology [1,10,11,12,13,14,15], the salient and distinctive clinical findings of the present patient mainly involved the craniofacial region (Table 2 and Figure 2A–C). In this regard, he manifested most of the craniofacial features proposed for PTHS clinical diagnosis (5/8) and only 2/17 (broad nasal tip and full lips) of CHUJANS (Table 1 and Table 2) [4].

**Table 2:** Clinical findings of this child compared with CHUJANS patients. References are cited from the most recent to the oldest.

Features	Conti 2023 [14] (*n* = 2) %	Kampmeier 2023 [15] (*n* = 23) %	Kaur 2021 [13] (*n* = 1) %	Craddock 2019 [12] (*n* = 10) %	Jansen 2018 [1] (*n* = 23) %	Webster 2016 [11] (*n* = 2) %	de Ligt 2012 [10] (*n* = 1) %	This Patient
ID	100%	91%	100%	100%	78%	100%	100%	+
DD	100%	96%	N.A.	100%	N.A.	100%	N.A.	+
Behavioral disturbances	N.A.	87%	100%	88%	78%	50%	N.A.	−
Upturned/short nose	100%	23%	100%	N.A.	68%	N.A.	N.A.	−
Thick alae nasi	N.A.	26%	N.A.	N.A.	68%	N.A.	N.A.	−
High forehead	N.A.	N.A.	100%	70%	67%	N.A.	N.A.	−
Large/fleshy earlobes	N.A.	61%	100%	40%	64%	100%	N.A.	−
Thick helices	N.A.	N.A.	100%	30%	50%	N.A.	N.A.	−
Thick earlobes	N.A.	N.A.	100%	10%	50%	N.A.	N.A.	−
Anormal eyebrows	N.A.	61%	0%	10%	59%	N.A.	100%	−
Anteverted nares	N.A.	52%	N.A.	40%	N.A.	N.A.	N.A.	−
Broad nasal tip	N.A.	17%	N.A.	20%	N.A.	N.A.	N.A.	+
Thin/full lips	100%	N.A.	0%	60%	36%	N.A.	100%	+
Up-turned upper lip	N.A.	N.A.	N.A.	N.A.	N.A.	50%	N.A.	−
Long/short/smooth/prominent philtrum	100%	30%	100%	40%	45%	50%	100%	−
Upslanting palpebral fissures/almond-shaped eyes	N.A.	13%	100%	50%	59%	N.A.	N.A.	−
Synophrys	100%	13%	0%	30%	59%	N.A.	N.A.	−
Deep set eyes	N.A.	17%	N.A.	N.A.	N.A.	50%	N.A.	−
Short/small nose	100%	30%	N.A.	20%	N.A.	50%	N.A.	−
Epicanthus	N.A.	9%	100%	10%	27%	N.A.	N.A.	−
Tapering fingers	N.A.	43%	0%	N.A.	76%	N.A.	100%	−
Clinodactyly 5th fingers	N.A.	30%	100%	50%	64%	N.A.	100%	+
Syndactyly 2/3 toe	N.A.	26%	N.A.	20%	30%	N.A.	N.A.	−
Brachydactyly	N.A.	30%	100%	N.A.	N.A.	N.A.	N.A.	+
Vision problems	N.A.	48%	100%	80%	65%	50%	100%	+
Fatigue	N.A.	9%	100%	90%	56%	N.A.	N.A.	−
Overweight/obesity	N.A.	70%	100%	20%	74%	100%	100%	−

Abbreviations: N.A. = not acquired; ID/DD = intellectual disability/developmental delay.

Regarding the other main *PHIP*-related findings, our patient displayed a very limited clinical overlap with the earlier patients. He showed some CHUJANS-unspecified characteristics, including vision problems, brachydactyly, and clinodactyly, while the most frequent features of the disorder, such as tapering fingers (>60%), overweight/obesity, and fatigue (60–40%), were not appreciable (Table 2).

## 3. Discussion

An additional patient with *PHIP*-associated neurodevelopmental disorder and unusual features due to a novel nucleotide change is illustrated here. 

Variants of this gene have recently been found in a specific ID–overweight syndrome with behavioral and craniofacial anomalies, including high forehead, full eyebrows/synophrys and fleshy ears, upturned nose with thick alae nasi, long philtrum, and thin lips. Other frequent phenotypic findings are represented by behavioral disturbances, malformed extremities, and other medical issues (muscular fatigue and visual problems) [1].

The present patient received an initial clinical diagnosis of a syndromic condition in the PTHS-like spectrum. Indeed, he displayed a distinctive craniofacial appearance, including wide nasal tip with flared alae nasi, wide mouth with full lips, cupid bow upper lip, widely spaced teeth, and full and prominent cheeks (Figure 2A–C and Table 1). In addition, he showed myopia and limb defects (brachydactyly, clinodactyly of 5th finger, broad halluces with camptodactyly, partial 2nd–3rd toe overriding, and bilateral 4th–5th feet clinodactyly) (Figure 2D and Table 2). The global phenotype was not suggestive of an overweight disorder with ID, comprising CHUJANS, which was unidentified as a possible diagnosis, highlighting the uncommon clinical presentation of this patient.

Of note, PTHS and overlapping disorders were not mentioned in any of the previously reported individuals both as probable diagnostic hypotheses and differential diagnoses.

This patient was a carrier of a novel *PHIP* variant, located in the C-terminal WD40 repeats, a fundamental domain mediating protein–protein interactions [1]. Indeed, a consistent part of previously disclosed transitions in CHUJANS impacts this protein site. The in silico analysis we performed supports the causative role of the detected variant. However, at this time, we cannot recognize a clear genotype–phenotype correlation for this individual. 

PHIP binds to the insulin receptor substrate 1 (IRS-1) that is required for the propagation of many of insulin’s biological effects. A recent paper demonstrated that a dominant-negative mutant of *PHIP* in fibroblasts specifically blocks transcriptional and mitogenic signals elicited by insulin [16]. These results are consistent with the hypothesis that PHIP represents a physiological protein ligand of the IRS-1 PH domain, playing an important role in insulin receptor-mediated mitogenic and metabolic signal transduction. On the other hand, insulin was shown to alter the expression of several Wnt pathway-related genes including *TCF4*, and the stimulatory effect of insulin on *TCF4* expression was largely demonstrated [17]. Indeed, several other studies have also reported the stimulatory effect of insulin or IGF-1 on β-cat nuclear localization and cat/TCF-mediated gene transcription [18,19,20].

Interestingly, experiments performed on a murine model of Angelman syndrome (AS, MIM#105830), a disease included in the differential diagnosis of PTHS, showed a behavioral rescue following acute systemic IGF-2 treatment [21]. These speculations postulate that facial similarities between our PTHS-like syndrome patient, harboring a *PHIP* variant, and other PTHS *TCF4*-mutated patients could have a possible link that lies in insulin signaling dysfunction.

Furthermore, in vitro data suggested that *PHIP* is co-expressed in human cells with another member of the TCF family—TCF12 (MIM*600480) [22]—giving the impression of a possible functional link between *PHIP* and *TCF4*. All these aspects need to be clarified in the future.

The potential limitations of our observations are represented by the unavailability of molecular studies better elucidating the biological basis of the unusual presentation of this child. 

The absence of clinical descriptions of other patients sharing the same genotype represents another limitation but also seems to be a challenge that stimulates the scientific community to new clinical descriptions.

## 4. Conclusions

The *PHIP*-associated disorder presents unusually in this child, for whom a clinical diagnosis of CHUJANS had not initially been considered, thus expanding the phenotype associated with the condition. This highlights a broad clinical spectrum within CHUJANS, which may include atypical presentations. *PHIP* variants should be investigated in patients who exhibit features resembling PTHS but are negative for mutations in *TCF4* and other genes linked to overlapping disorders. 

At present, our observations are limited and represent only preliminary data; this patient’s clinical presentation aligns with a PTHS-like disorder. 

Further descriptions of additional patients and future molecular studies are necessary. Nevertheless, clinicians should be informed of this rare presentation, which contributes to a broader understanding of the associated phenotype.

## Figures and Tables

**Table 1:** PTHS main diagnostic features according to the PTHS International Consensus [4]. The clinical findings of this patient are indicated.

Main PTHS Features	This Patient
Severe ID	+ (moderate)
Microcephaly	+
Narrow forehead	−
Thin lateral eyebrows	−
Wide nasal bridge/ridge/tip	+
Flared nasal alae	+
Full cheeks/prominent midface	+
Wide mouth/full lips/cupid bow upper lip	+
Thickened/overfolded helices	−
Breathing regulation anomalies (intermittent hyperventilation and/or apnea)	−
Myopia	+
Constipation	not acquired.
Slender fingers and/or abnormal palmar creases	−
Unstable gait	+ (moderate)

## Data Availability

Data regarding this study are available from the corresponding author upon reasonable request, due to privacy reasons.
